# Biphasic Effects of FGF2 on Adipogenesis

**DOI:** 10.1371/journal.pone.0120073

**Published:** 2015-03-19

**Authors:** Sooho Kim, Chihoon Ahn, Naeun Bong, Senyon Choe, Dong Kun Lee

**Affiliations:** 1 Laboratory of Genome to Drug Medicine, joint Center for Biosciences, Incheon, Korea; 2 Laboratory of Synthetic Biology, joint Center for Biosciences, Incheon, Korea; Faculty of Biology, SPAIN

## Abstract

Although stem cells from mice deficient of FGF2 have been reported to display enhanced capacity for adipogenesis, the literature using in vitro cell culture system has so far reported conflicting results on the role of FGF2 in adipogenesis. We here demonstrate that FGF2, depending on concentration, can function as either a positive or negative factor of *in vitro* adipogenesis by regulating activation of the ERK signaling pathway. FGF2 at concentrations lower than 2 ng/ml enhanced *in vitro* adipogenesis of human adipose-derived stem cells (hASCs). However, FGF2 at concentrations higher than 10 ng/ml was able to suppress adipogenesis by maintaining sustained phosphorylation of ERK and function as a dominant negative adipogenic factor toward BMP ligands. Expression levels of FGF2 in the fat tissues from high fat diet induced obese C57BL/6 mice were lower than those from normal chow diet mice, indicating that expression levels of FGF2 in the fat tissues might be in reverse correlation with the size of fat tissues. Our observation of concentration dependent biphasic effect as well as dominant negative effect of FGF2 on adipogenesis provides a mechanistic basis to understand roles of FGF2 in adipogenesis and development of fat tissues.

## Introduction

Adipogenesis, which determines differentiation of fibroblast-like mesenchymal precursor stem cells into lipid-laden and insulin-responsive adipocytes, requires networks of signaling pathways [1,2,3] and extracellular factors [4]. The mitogen activating protein kinase (MAPK) signaling pathway has been identified to regulate differentiation of stem cells into adipocytes. Activation of ERK is necessary for proliferation of preadipocytes and initiation of differentiation [5]. However, phosphorylation of PPARγ by ERK suppresses PPARγ activity, and ERK needs to be shut-off to proceed with maturation of adipocytes [6,7]. Mouse embryo fibroblasts from ERK1^−/−^ mice displayed impaired adipogenesis and ERK1^−/−^ mice challenged with high fat diet are resistant to obesity [8]. Activity of ERK during adipogenesis is regulated by two proteins, DUSP-1 and AE binding protein (AEBP)-1. Expression of dual specificity protein phosphatase-1 (DUSP-1), which inactivates ERK, is up-regulated in mature adipocytes [9]. Conversely, AEBP-1, which binds to ERK and protects from phosphatases, is down-regulated in mature adipocytes [6].

Growth factors, hormones, and morphogens have also been reported to regulate adipogenesis [4]. Bone morphogenetic proteins (BMPs) play a distinct role in modulating adipogenesis depending on the types and concentrations of BMPs [10]. Studies indicate that BMP-2, -4, -6, -7, -9, -12, -13, and -14 promote *in vitro* adipogenesis of mesenchymal stem cells among the 14 BMPs tested [10]. In addition, BMP-7 and -8b have been reported to promote differentiation into brown adipocytes [11,12]. Mice deficient with BMP-4 exhibited enlarged white adipocytes and impaired insulin sensitivity, indicating a role of BMP-4 in brown adipogenesis [13]. We recently reported that BMP-9 enhanced brown adipogenesis and suppressed high fat diet induced obesity [14].

FGF family ligands have been reported to determine embryogenesis as a morphogenic factor. Spatial and temporal expression of FGF family ligands plays a key role in development of organs [15]. Studies using chick embryonic limb indicated that proximo-distal gradients of FGF2 determined patterning of embryonic limb buds [16,17]. Another study using Xenopus demonstrated that FGF2 gradient determined the anteroposterior axis of the central nervous system with lower doses for anterior parts and higher doses for posterior parts of the central nervous system [18].

In addition to regulating embryogenesis, FGF ligands regulate proliferation and differentiation of stem cells into various types of cells [15,19]. FGF1 has been reported to promote both proliferation and differentiation of preadipocytes [20]. A recent study has demonstrated that FGF1 plays a role in adipose tissue remodeling as well as metabolic homeostasis and insulin sensitization [21,22]. FGF2 has been proposed to be required for self-renewal of human adipose tissue derived stem cells (hASC) [23]. A recent study demonstrated that FGF2 suppresses differentiation of mesenchymal stem cells by inducing Twist2 and Sprouty4 (Spry4) [24]. Bone marrow stem cells from mice deficient of FGF2 displayed enhanced capacity for adipogenesis [25], indicating FGF2 as a negative adipogenic factor. However, the literature using in vitro cell culture system has so far reported conflicting results on the role of FGF2 in adipogenesis [26,27].

Since each literature reporting discrepancies on the role of FGF2 in *in vitro* adipogenesis used different concentrations of FGF2, ranging from 3 ng/ml [27] to 20 ng/ml [24], to modulate adipogenesis, we hypothesized that FGF2 might display biphasic effects on adipogenesis depending on concentrations. Thus, we carried out in vitro adipogenesis using hASCs to investigate concentration-dependent effects of FGF2 on adipogenesis and identified mechanisms underlying biphasic effect of FGF2 on adipogenesis. Expression levels of FGF2 in fat tissues from normal and obese mice were also analyzed to determine the relationship between high fat diet-induced obesity and expression levels of FGF2 in fat tissues.

## Materials and Methods

### Recombinant protein ligands

Recombinant human FGF1, FGF2, and BMP-2 contained the first methionin and mature forms of each ligand. MB109 is a recombinant derivative of human BMP-9 containing a methionine residue in front of the mature form of human BMP-9 (Ser338-Arg429). Recombinant ligands were purchased from joint Protein Central (Incheon, Korea). BMP-2 and MB109 were reconstituted in 5 mM HCl (concentrations ≥ 0.8 mg/ml) and diluted in cell culture medium just before use. FGF ligands (1 mg/ml) in PBS plus 0.1 mM dithiothreitol were filtered with a 0.2 micron filter, aliquated, and stored in −70°C. The signaling activities of the recombinant proteins were routinely determined by cell-based assay luciferase activity.

### Adipogenesis of hASCs

Human Adipose-derived Stem Cells (hASCs) purchased from Invitrogen (Carlsbad, USA) were grown in growth medium (MesenPro RS supplemented with 10 μg/ml Gentamicin and 2% FBS) to be confluent, and treated with various concentrations of ligands in the growth medium for 1 day, and then induced for adipogenic differentiation for 7 days in the differentiation medium (growth medium plus 500 μM IBMX, 1 μM dexamethasone, 850 nM insulin, 125 nM indomethacin, 1 nM T3, and 1 μM rosiglitazone).

### Real-time PCR

Complementary DNAs were synthesized from purified RNA using Premium Express cDNA synthesis system (LeGene Biosciences, USA) according to the manufacturer’s instruction. For GC pair rich human genes, 3% DMSO was added to cDNA synthesis reaction mixtures. Sequences of primers for aP2, C/EBPα, PPARγ, DUSP1, Spry4, and cyclophillin are shown in Table 1. Abundance of mRNA of interest in each sample was determined by the ΔCτ (cycle threshold), the difference between the Cτ values for gene of interest and cyclophilin.

**Table 1:** Sequences of primers for real time-PCR.

gene	forward	reverse
**ap2**	5'CATGTGCAGAAATGGGATGG3'	5'AACTTCAGTCCAGGTCAACG3'
**C/EBP α**	5'TGGACAAGAACAGCAACGAG3'	5'TCATTGTCACTGGTCAGCTC3'
**cyclophilin**	5'CGAGGAAAACCGTGTACTATTAG3'	5'TGCTGTCTTTGGGACCTTG3'
**DUSP1**	5'ACCACAAGGCAGACATCAG3'	5'AAGGTAAGCAAGGCAGATGG3'
**PPARγ**	5'GAGCCCAAGTTTGAGTTTGC3'	5'GCAGGTTGTCTTGAATGTCTTC3'
**Spry4**	5'CACTCACCATCCTACCCATTG3'	5'CAGGCTAGGGTTGTCTATGTAG3'

### Preparation of cell lysates and Western blotting analysis

Cells treated as indicated were lysed by adding high salt cell lysis buffer (20 mM Tris-HCl/pH 7.5, 1 mM EDTA, 1 mM EGTA, 1% Triton X-100, 1 mg/ml leupeptin, 2.5 mM sodium pyrophosphate, 1 mM beta-glycerophosphate, 1 mM Na_3_VO_4_, 0.3 M NaCl, 0.5 mM phenylmethanesulfonyl fluoride) plus phosphatase inhibitor cocktail (Cell Signaling Technology), and centrifuged at 12,000 x *g* for 5 min at 4°C. Total cell lysates were separated by SDS-PAGE and transferred onto nitrocellulose membranes. Proteins on membranes were incubated with antibodies against ERK or phosphorylated form of ERK (Cell Signaling Technology) and processed for Western blot analyses using enhanced chemiluminescence detection kit.

### Animal experiment

All animal experimental procedures were performed in accordance with protocols approved by the Gachon University Institutional Animal Care and Use Committee (approval number LCDI-2012-0080). C57BL/6 (male, 8 wk old) mice were randomly assigned to normal chow diet (NC) and high fat diet (HF) groups (HF/sham, HF/MB109, 4 mice per cage, 8 mice per group). Lard provides 60% energy of the HF diet (Research Diets). Mice were housed in a temperature and humidity controlled specific pathogen free facility with 12 h dark-light cycles. Food consumption and body weights of mice were recorded every week. Each mouse received intraperitoneal injection of vehicle (PBS) or 100 μg/kg twice per week of recombinant human MB109 for 8 wks. Mice were euthanized with carbon dioxide around 9 weeks after starting high fat diet.

### Statistical Analysis

Data are presented as mean ± standard deviation (SD). Statistical comparison of data was determined using one-way analysis of variance, followed by the Dunnett post-hoc adjustment.

## Results

### Biphasic effects of FGF2 on expression of aP2 and C/EBP

**Fig 1:**
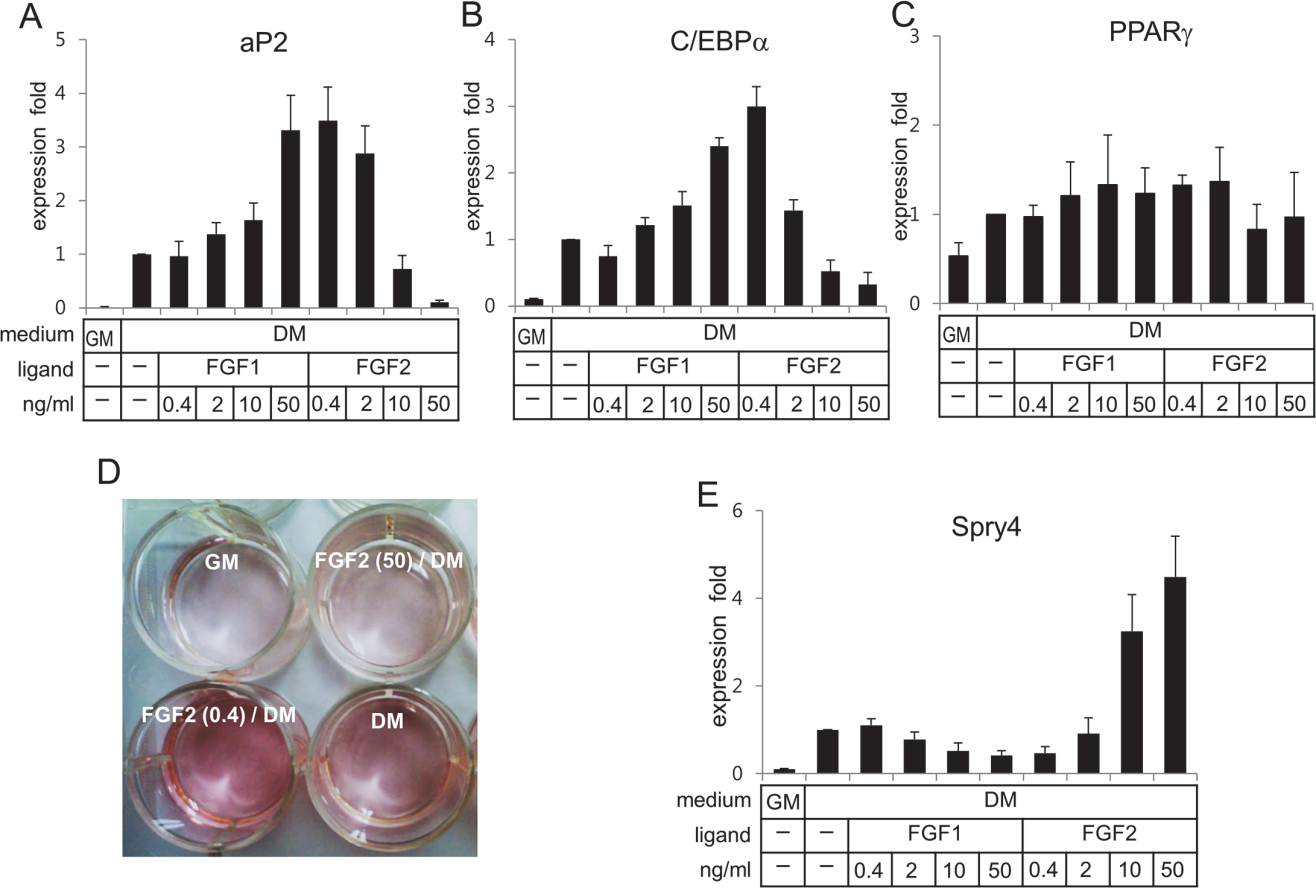
While FGF1 enhances adipogenesis of hASCs in a dose-dependent manner, FGF2 displays biphasic effects on adipogenesis. Human ASCs were pre-conditioned in the growth medium (GM) with various concentrations of ligands for 1 day, washed with PBS, treated in the differentiation medium (DM) for 6 days, and subjected to extraction of total RNA. Analyses of expression of aP2 (A), C/EBPα (B), and PPARγ (C) genes were carried out using real-time PCR with cyclophilin as an internal control. (D) Human ASCs were differentiated for 2 weeks and subjected to oil red O staining. (E) Human ASCs were pre-conditioned in the GM with the ligands for 1d, washed, treated with the DM for 16 h, and subjected to extraction of total RNA. Analysis of Spry4 was carried out using real-time PCR with cyclophilin as an internal control. Average values of each gene expression in the DM without ligands were calculated as 1 for statistical analysis. Results are presented as means ± SD.

In order to analyze dose-dependent effects of FGF1 and FGF2 on adipogenesis, human ASCs were preconditioned with different concentrations of the FGF ligands in the growth medium for 1 day and subsequently grown in the differentiation medium without the FGF ligands for 7 days. Whereas FGF1 enhanced expression of adipocyte protein 2 (aP2), an adipogenic marker protein, in a dose dependent manner, FGF2 enhanced it at concentrations 0.4 ~ 2 ng/ml but suppressed at concentrations 10 ng/ml and higher (Fig. 1A). Effects of FGF2 on aP2 mRNA expression were inversely proportional to concentrations of FGF2. Since induced expression of PPARγ and C/EBPα has been reported to be required for expression of adipogenic genes, dose-dependent effects of the FGF ligands on expression of PPARγ and C/EBPα were analyzed. FGF1 enhanced mRNA expression of C/EBPα in a dose-dependent manner (Fig. 1B). Again, FGF2 enhanced it at concentrations 2 ng/ml and lower, but suppressed at concentrations 10 ng/ml and higher (Fig. 1B). However, dose-dependent effects of the FGF ligands on mRNA expression of PPARγ were not as evident as those on C/EBPα mRNA expression (Fig. 1B and C). The mRNA expression patterns of aP2 by these FGF ligands were matched with those of C/EBPα but not with those of PPARγ (Fig. 1A vs 1B or 1C). Oil red O staining analysis also confirmed that FGF2 enhanced adipogenesis at 0.4 ng/ml and suppressed adipogenesis at 50 ng/ml (Fig. 1D).

Since Spry4 has been identified as an antagonist of FGF signaling and linked to inhibition of the receptor-transduced MAP kinase signaling pathway [24], dose-dependent effects of the FGF ligands on expression of Spry4 were analyzed. Expression levels of Spry4 were inversely proportional to doses of FGF1 (Fig. 1E), which is consistent with the mRNA expression profiles of aP2 and C/EBPα. Conversely, FGF2 at a concentration of 0.4 ng/ml reduced Spry4 expression, but markedly enhanced it at concentrations of 10 ng/ml or higher in a dose-dependent manner (Fig. 1E).

### Sustained activation of ERK by high concentration of FGF2

**Fig 2:**
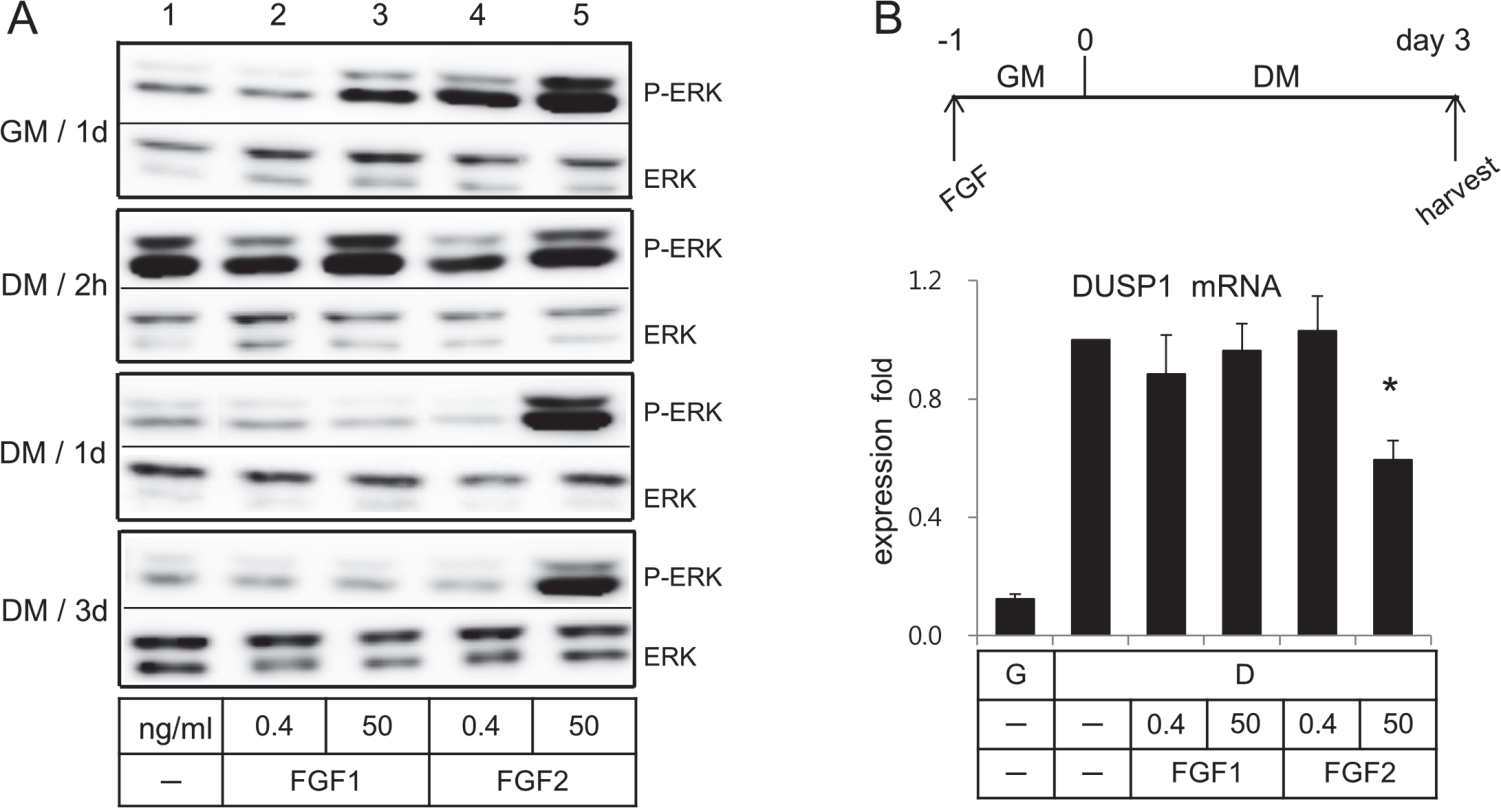
FGF2 at 50 ng/ml displayed sustained phosphorylation of ERK, while FGF1 displayed phosphorylation and dephosphorylation patterns of ERK upon treatment of cells with the differentiation medium. (A) Human ASCs were pre-conditioned in the GM with the ligands for 1d, washed, treated with the differentiation medium as indicated, and subjected to extraction of cell lysates. (B) Human ASCs were treated as indicated in the panel B and subjected to extraction of total RNA. Analysis of expression of DUSP1 was carried out using real-time PCR with cyclophilin as an internal control. Average values of DUSP1 gene expression in the DM without ligands were calculated as 1 for statistical analysis. Results are presented as means ± SD.

Preconditioning of cells in the growth medium with low (0.4 ng/ml) concentration of FGF1 showed little effect on phosphorylation patterns of ERK (lane 1 *vs* lane 2 in Fig. 2A), but that with high (50 ng/ml) concentration slightly enhanced a phosphorylation degree of ERK. However, preconditioning of cells in the growth medium with FGF2 enhanced a phosphorylation degree of ERK in a dose dependent manner even before treatment with the differentiation medium (DM) (lanes 4 and 5 in Fig. 2A). Consistent with the published reports [5,6], phosphorylation of ERK was observed immediately after treatment of cells with the differentiation medium (see lane 1 of DM/1h in Fig. 2A). Since ERK was minimally phosphorylated when cells were preconditioned with FGF1 in the growth medium (GM), ERK was remarkably phosphorylated when cells preconditioned with FGF1 were treated with the differentiation medium (lanes 2 and 3 of DM/1h in Fig. 2A). Nevertheless, treatment of cells with the differentiation medium did not further enhance phosphorylation degrees of ERK by FGF2 (lanes 4 and 5 in GM/1d *vs* DM/1h).

Dephosphorylation of ERK was observed at 1 day after treatment of hASCs with the differentiation medium (lane 1 of DM/1d in Fig. 2A). Dephosphorylation of ERK was observed in cells preconditioned with both low and high concentrations of FGF1. With FGF2, however, it was observed in cells preconditioned only with the low concentration of FGF2. Cells preconditioned with the high concentration (50 ng/ml), we observed sustained phosphorylation of ERK even 3 days after treatment with the differentiation medium (lane 5 in Fig. 2A).

DUSP1, which regulate phosphorylation of ERK, has been reported to be up-regulated in mature adipocytes. We therefore analyzed dose-dependent effects of the FGF ligands on DUSP1 mRNA expression. Incubation of hASCs in the differentiation medium for 3 days enhanced DUSP1 mRNA expression (Fig. 2B). Preconditioning of hASCs with either the low or high concentrations of FGF1 showed little effect on DUSP1 mRNA expression patterns (Fig. 2B). Preconditioning with the low concentration of FGF2 did not change an expression level of DUSP1, however, the high concentration of FGF2 suppressed DUSP1 expression (Fig. 2B).

### Dominant negative effect of FGF2 at 50 ng/ml on in vitro adipogenesis

**Fig 3:**
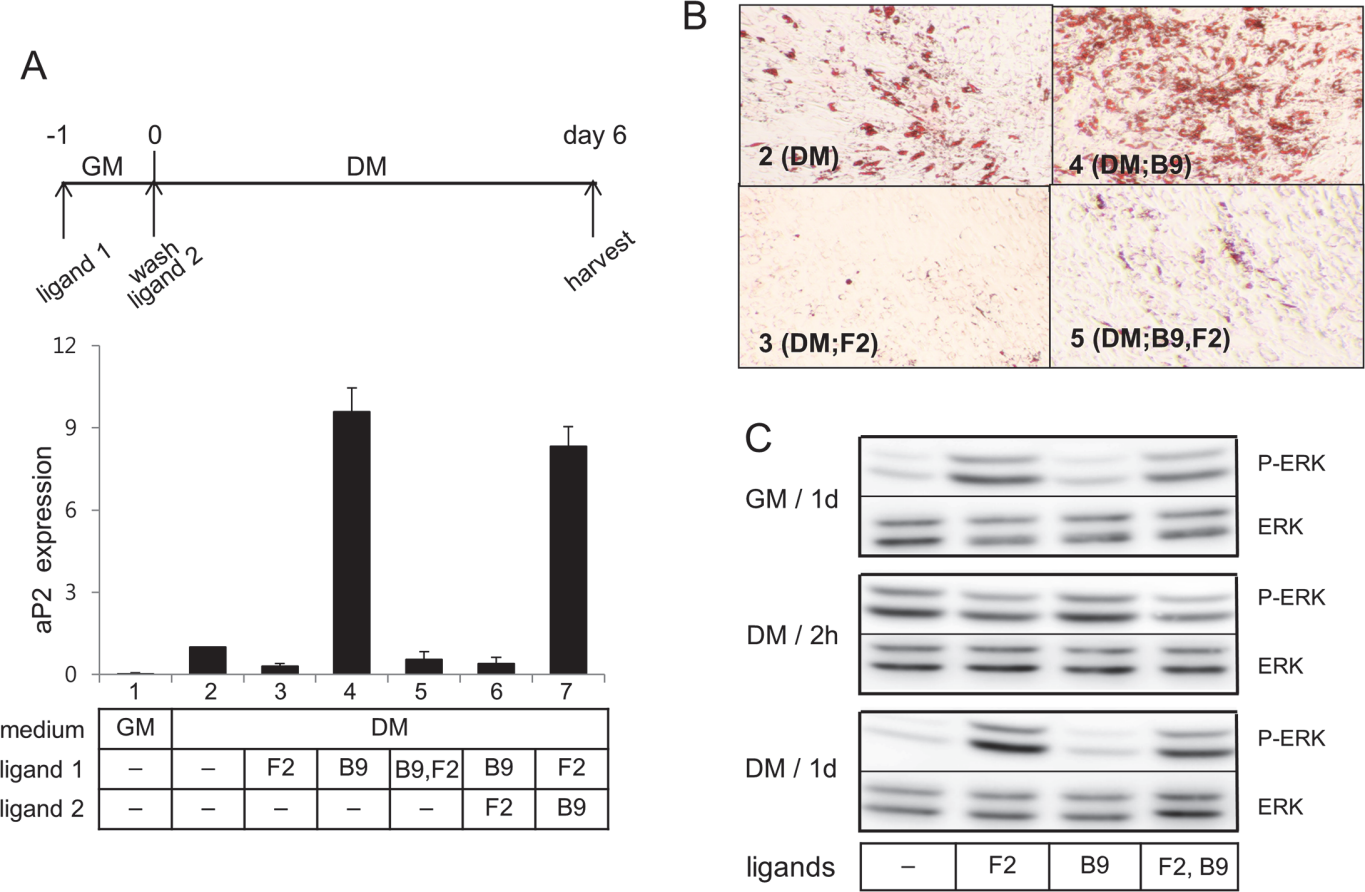
FGF2 at 50 ng/ml can function as a dominant negative adipogenic factor toward BMP-9. (A) Human ASCs were treated as indicated in the panel A and subjected to extraction of total RNA. Analysis of expression of aP2 was carried out using real-time PCR with cyclophilin as an internal control. Average values of aP2 gene expression in the DM without ligands were calculated as 1 for statistical analysis. Results are presented as means ± SD. (B) Human ASCs were treated as described in the panel A and differentiated for 2 weeks and subjected to oil red O staining (final magnification X40). (C) Human ASCs were pre-conditioned in the GM with the ligands for 1d, washed, treated with the differentiation medium as indicated, and subjected to extraction of cell lysates.

BMP-9 (100 ng/ml) alone markedly enhanced aP2 mRNA expression (lane 4 in Fig. 3A) and induced differentiation into lipid laden adipocytes (panel 4 in Fig. 3B). To our surprise, FGF2 at concentration 50 ng/ml, when incubated together with BMP-9, was able to suppress aP2 expression induced by BMP-9 (lane 5 in Fig. 3A), indicating that FGF2 at 50 ng/ml was able to function as a dominant negative adipogenic factor. The dominant negative effect of FGF2 at 50 ng/ml on adipogenesis was also confirmed with oil red O staining (panel 5 in Fig. 3B). As expected, subsequent incubation of cells with FGF2 (50 ng/ml) following incubation with BMP-9 (100 ng/ml) also suppressed adipogenic effect of BMP-9. When cells were incubated with FGF2 (50 ng/ml) for 1 day in the growth medium, washed, and incubated with BMP-9 (100 ng/ml), the suppression did not occur so that cells markedly express aP2 mRNA (lane 7 in Fig. 3A) and differentiate into adipocytes (panel 7 in Fig. 3B). Dominant negative effect of FGF2 (50 ng/ml) on the adipogenesis was also observed with BMP-2 (S1 Fig.). On the other hand, FGF2 at low concentration (0.4 ng/ml), when incubated together with BMP-2, did not show effects on aP2 expression induced by BMP-2 (S1 Fig.).

Western blot analysis displayed that BMP-9 caused little change in the phosphorylation patterns of ERK by induction of differentiation. While preconditioning of cells with BMP-9 alone caused dephosphorylation of ERK at 1 day after induction of differentiation, preconditioning with BMP-9 and FGF2 together did not cause dephosphorylation of ERK (panel C in Fig. 3C).

### Reduced FGF2 mRNA expression in the obese mouse fat tissue

**Fig 4:**
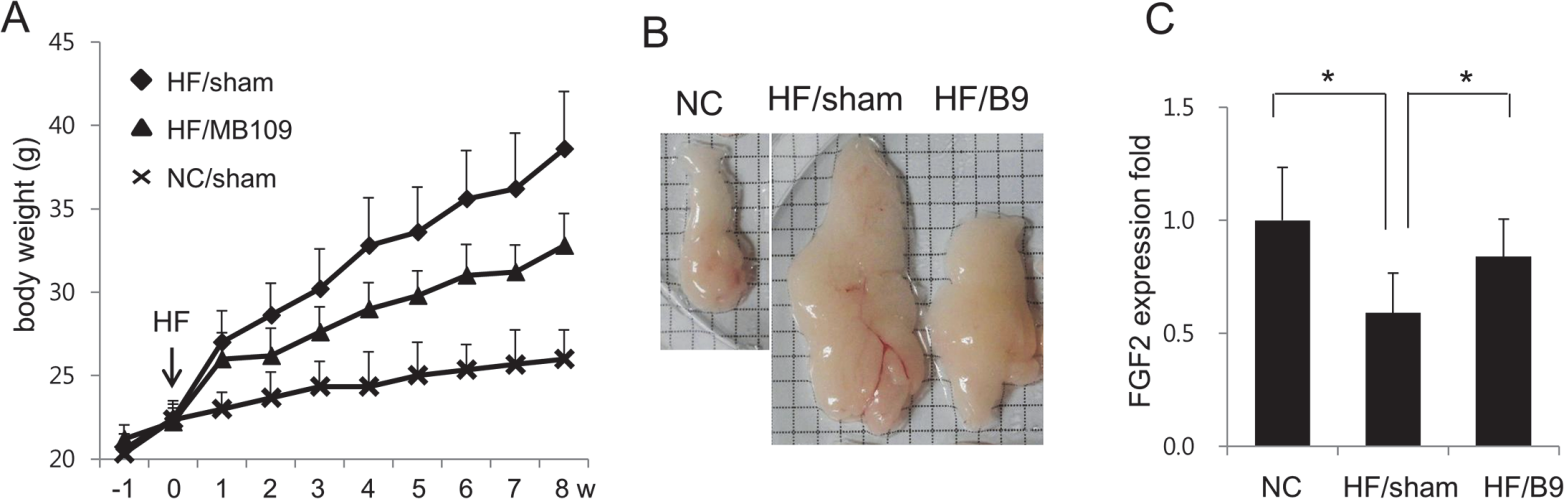
High fat diet induced obesity decreased FGF2 expression level in the fat tissues. (A) Body weight changes of C57BL/6 mice fed with normal chow diet (NC, n = 8) or high fat diet (HF, n = 8) were observed for 9 wks. Intraperitoneal injection of vehicle (PBS), or MB109 (200 μg/kg/wk) were performed once a week for 8 wks. (B) Representative images of epididymal fat tissues are shown. (C) Real-time PCR was carried out with cyclophilin as an internal control. An average value of FGF2 expression in the epididymis fat tissue of NC/sham mice was calculated as 1 for statistical analysis. Results are presented as means ± SD. * *p* < 0.05 *vs* sham control.

In order to analyze relationship between FGF2 expression patterns and high fat diet-induced obesity, FGF2 mRNA expression levels in the epididymis adipose tissues from mice fed with normal chow diet or high fat diet were determined. Since we previously reported that intraperitoneal injection of BMP-9 (200 μg/kg/wk) suppressed high fat diet-induced obesity [14], we also determined if BMP-9 injection changed expression levels of FGF2 in the adipose tissues. As expected, periodical injection of BMP-9 suppressed weight gaining of mice fed with high fat diet (Fig. 4A). Fat tissues from mice injected with BMP-9 and fed with high fat diet were larger than those from mice fed with normal chow diet, but smaller than those from the high fat sham control group (Fig. 4B). FGF2 mRNA expression levels in the epididymis fat tissues from mice fed with high fat diet were lower than those from mice fed with normal chow diet (Fig. 4C). Since adipocytes are the only source of FGF2 mRNA expression in the adipose tissues [28], results indicated that adipocytes in the obese epididymis fat tissues produced lower levels of FGF2 mRNA than those in the normal fat tissues. Systemic injection of BMP-9 decreased the sizes of epididymis fat tissues and enhanced FGF2 mRNA expression levels in the adipocytes (Fig. 4B and C).

## Discussion

Mesenchymal stem cells from FGF2^−/−^ mice displayed suppressed osteogenesis and enhanced adipogenesis, indicating FGF2 as a negative adipogenic factor [25]. FGF2 at concentrations 10 and 20 ng/ml has been reported to stimulate activation of ERK and enhance *in vitro* osteogenesis of mouse C3H10T1/2 cells [29]. Contrary to the effect of FGF2 on osteogenesis, we observed that enhanced phosphorylation of ERK by FGF2 at 10 ng/ml or higher concentration suppressed adipogenesis. However, our study also showed that FGF2 at 2 ng/ml or lower concentration enhanced adipogenic gene expression. Concentration dependent biphasic effect of FGF2 on osteogenesis has not been observed. Results in this study indicating concentration-dependent biphasic effect of FGF2 on adipogenic gene expression would provide mechanisms to explain contradictory reports of FGF2 in *in vitro* adipogenesis [26,27].

According to the publication by Lazar group [30], adipogenesis consists of 4 stages; proliferation of preadipocytes (stage I), PPAR activation (stage II), retinoic acid insensitive stages (stages III, IV). Phosphorylation of ERK is required for proliferation of preadipocytes and initiation of adipogenesis (stage I). However, ERK should be deactivated before the stage II, which requires activation of PPAR. Our study showed that sustained phosphorylation of ERK by the high concentration of FGF2 inhibited expression of adipogenic genes, presumably due to phosphorylation and consequent inactivation of PPARγ by ERK. Enhanced osteogenesis by FGF2 at concentrations which suppress adipogenesis might be explained by the report demonstrating enhanced osteogenesis and suppressed adipogenesis by silencing of PPARγ in hASC [31]. Further studies are necessary to elucidate detailed mechanisms by which stimulation of the ERK pathway by the high concentration of FGF2 leads to stimulation of osteogenesis but suppression of adipogenesis. A recent study reported that adipogenic inhibitor Pref-1 activated ERK and inhibited adipogenesis [32]. Since DUSP1 mRNA expression was reduced not by the low concentration but by the high concentration of FGF2, sustained phosphorylation of ERK at the high concentration of FGF2 might be partly attributed to the reduced expression of DUSP1 expression.

**Fig 5:**
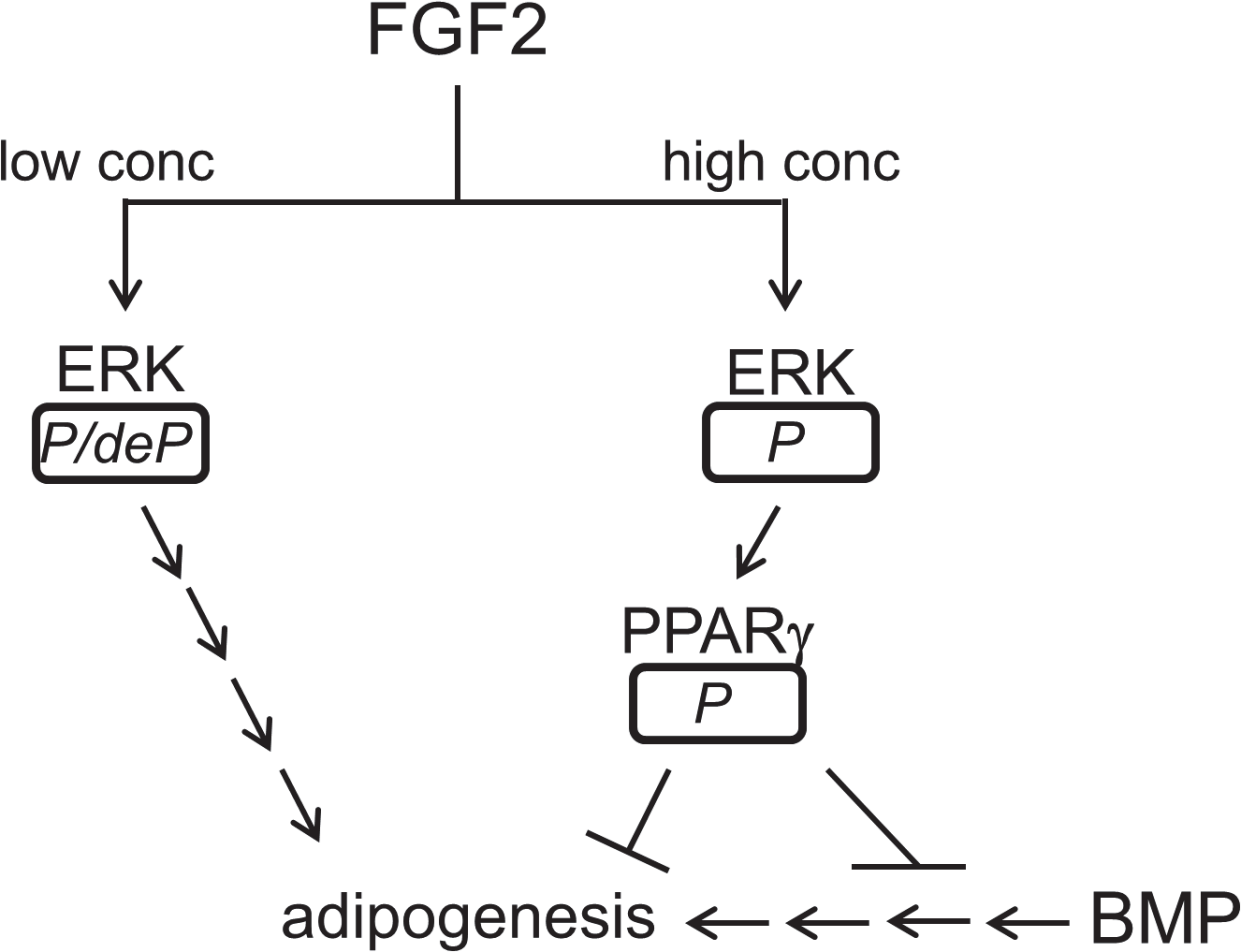
Interrelationship of FGF2/ERK/PPARγ/BMP9 on adipogenesis is shown in the figure. While an italic *P/deP* represents phosphorylation and subsequent dephosphorylation of ERK, an italic *P* represents sustained phosphorylation of ERK or PPARγ. Arrows indicate activation or stimulation of steps in directions of arrowheads. Crossbars indicate suppression of steps.

Since interplay of various growth and morphogenic factors modulates differentiation and tissue development, suppression effect of FGF2 at 50 ng/ml on adipogenesis was analyzed in combination of adipogenic inducer BMP-2 and -9. Simultaneous incubation of hASCs with 50 ng/ml FGF2 and 150 ng/ml BMP ligands suppressed adipogenic induction of the BMP ligands, suggesting dominant negative effect of FGF2 at the high concentration on adipogenesis. Presence of BMP ligands was not able to change sustained phosphorylation of ERK by FGF2 (50 ng/ml). However, removing FGF2 from the culture medium and further incubation of hASCs with BMP-9 allowed hASCs to undergo adipogenesis, since FGF2 was not able to suppress adipogenic induction of BMP-9 by removal of FGF2 and sequential addition of BMP-9. This result indicates that temporal expression of FGF2 and BMP ligands may play a critical role in adipogenesis. Interrelationship of FGF2/ERK/PPARγ/BMP on adipogenesis is shown in Fig. 5.

A study reporting FGF2 mRNA expression throughout *in vitro* adipogenesis demonstrated that FGF2 was strongly expressed in preadipocytes but its expression was markedly reduced during adipogenesis [33]. Our study indicating enhanced *in vitro* adipogenesis by the low concentration of FGF2 is in line with marked reduction of FGF2 in mature adipocytes. In addition, analysis of the mouse epididymis fat tissues indicated that expression levels of FGF2 mRNA were lower in the epididymis fat tissues from high fat diet-induced obese mice. Suppression of FGF2 expression in adipocytes appears to be necessary for a high fat diet-mediated increase in the size and number of adipocytes in the epididymis adipose tissues. BMP-9, which has been reported to enhance brown adipogenesis and suppress obesity [14], may counteract the high fat diet-mediated suppression of FGF2 expression in the epididymal adipocytes. Enhanced expression of FGF2 in the adipose tissues by BMP-9 injection might underlie the mechanism by which BMP-9 suppressed high fat diet-induced obesity. Results in the study suggest that temporal and spatial expression of FGF2 and BMP ligands may play a critical role in adipogenesis and development of adipose tissues.

## Supporting Information

S1 Fig While high concentration FGF2 (50 ng/ml) suppressed BMP-2 mediated adipogenesis, low concentration FGF2 (0.4 ng/ml) did not further enhanced BMP-2 mediated adipogenesis. Human ASCs were pre-conditioned in the growth medium (GM) with various concentrations of FGF2 or 150 ng/ml BMP-2 for 1 day, washed with PBS, treated in the differentiation medium (DM) for 6 days, and subjected to extraction of total RNA. Analysis of expression of aP2 gene was carried out using real-time PCR with cyclophilin as an internal control. Results are presented as means ± SD; n (numbers of experiments performed) = 3.
(PDF)
