# Supplementary figures and images for: Biphasic Effects of FGF2 on Adipogenesis
Source: PLoS One. 2015 Mar 19;10(3):e0120073. doi: 10.1371/journal.pone.0120073 (PMC4366188; doi:10.1371/journal.pone.0120073)

S1\_Fig

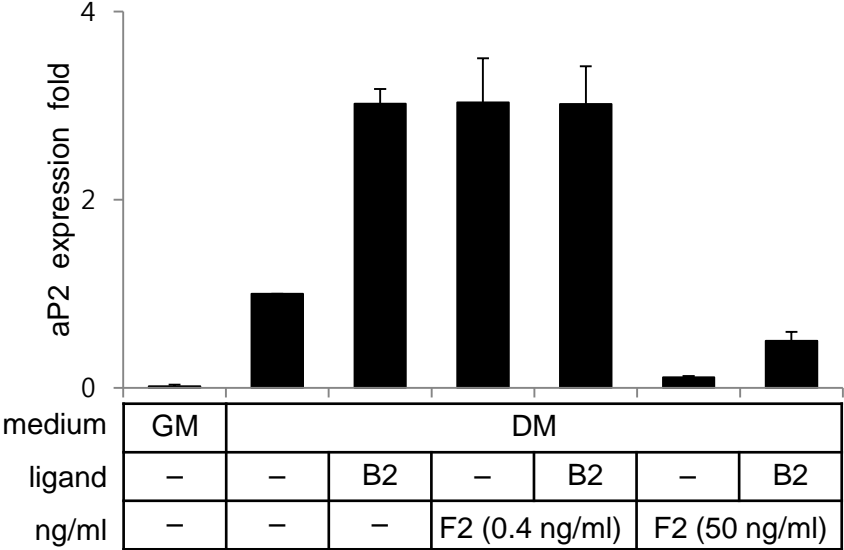

Supplement: S1 Fig — Human ASCs were pre-conditioned in the growth medium (GM) with various concentrations of FGF2 or 150 ng/ml BMP-2 for 1 day, washed with PBS, treated in the differentiation medium (DM) for 6 days, and subjected to extraction of total RNA. Analysis of expression of aP2 gene was carried out using real-time PCR with cyclophilin as an internal control. Results are presented as means ± SD; n (numbers of experiments performed) = 3. (PDF) [file pone.0120073.s001.pdf]
